# 5G Positioning: An Analysis of Early Datasets

**DOI:** 10.3390/s23229222

**Published:** 2023-11-16

**Authors:** Chiara Pileggi, Florin Catalin Grec, Ludovico Biagi

**Affiliations:** 1 DICA Politecnico di Milano, Piazza Leonardo Da Vinci 32, 20133 Milano, Italy; chiara.pileggi@polimi.it; 2 European Space Agency, Keplerlaan 1, 2201AZ Noordwijk, The Netherlands; florin-catalin.grec@esa.int

**Keywords:** 5G, new radio, GNSS, network-based positioning, hybrid positioning, time of arrival

## Abstract

Global Navigation Satellite Systems (GNSSs) are nowadays the prevailing technology for positioning and navigation. However, with the roll-out of 5G technology, there is a shift towards ‘hybrid positioning’: indeed, 5G time-of-arrival (*ToA*) measurements can provide additional ranging for positioning, especially in environments where few GNSS satellites are visible. This work reports a preliminary analysis, the processing, and the results of field measurements collected as part of the GINTO5G project funded by ESA’s EGEP programme. The data used in this project were shared by the European Space Agency (ESA) with the DICA of Politecnico di Milano as part of a collaboration within the ESALab@PoliMi research framework established in 2022 between the two organizations. The *ToA* data were collected during a real-world measurement campaign and they cover a wide range of user environments, such as indoor areas, outdoor open sky, and outdoor obstructed scenarios. Within the test area, eleven self-made replica 5G base stations were set up. A trolley, carrying a self-made 5G receiver and a data storage unit, was moved along predefined trajectories; the trolley’s accurate trajectories were determined by a total station, which provided benchmark positions. In the present work, the 5G data are processed using the least squares method, testing and comparing different strategies. Therefore, the primary goal is to evaluate algorithms for position determination of a user based on 5G observations, and to empirically assess their accuracy. The results obtained are promising, with positional accuracy ranging from decimeters to a few meters in the worst cases.

## 1. Introduction

Global Navigation Satellite Systems (GNSSs) have been the dominant technology for positioning, navigation, and timing for decades [1,2]. Our smartphones seamlessly fuse GNSS data with readings from other sensors to improve position estimation accuracy; the deployment of 5G technology across the world is also expected to open new opportunities in the domain of positioning, not just communication. Firstly, 5G networks can broadcast GNSS corrections for improved accuracy. To continue, 5G can augment GNSSs by providing additional range measurements in environments where only a few satellites are visible. The accurate determination of location information is a strategic byproduct of 5G cellular networks [3,4,5], as it has the potential to benefit numerous commercial applications, from individual to public services. This covers application domains such as transportation, public safety, retail, and healthcare. The integration with GNSSs [6,7,8] becomes relevant in urban areas, where reliance on GNSSs alone could be challenging [9]. Compared to previous mobile generations such as LTE, 5G technology features a new radio-access technology called new radio (NR), which offers several advantages for precise positioning. Designed by the 3rd Generation Partnership Project (3GPP), 5G NR is engineered to meet a variety of performance metrics, serving enhanced mobile broadband (eMBB), ultra-reliable low-latency communication (URLLC), and massive machine-type communication (mMTC) applications [10]. Next, we present the most important key enablers for accurate positioning. It is important to realize that while this list is not directly pertinent to the following discussion of our experiment, it could be useful as a summarized introduction to the technological aspects.

New positioning reference signals: to enable more accurate positioning than LTE with release 16 [11], the 3GPP updated the positioning reference signal (PRS) and sounding reference signal (SRS), providing the downlink (DL) and uplink (UL) signals, respectively [12]. Traditional signals such as channel state information reference signals (CSI-RSs) and synchronization signals (SSs) have limitations that make them less suitable for positioning: interference caused by adjacent cells reduces their ability to detect a sufficient number of neighboring 5G base stations; furthermore, the signals of neighboring cells overshadow those from distant ones (near-field effect), hindering the detection of the latter. The new signals are specifically designed to improve audibility thanks to the muting concept [13]: multiple base stations can transmit PRS in a coordinated manner by literally ’muting’ the less relevant PRS transmissions to avoid interference.

New positioning methods: 5G can provide different measurements that are related to the position of the user equipment (UE). In summary, these measurements can be based either on angles or on distances [14,15]. To elaborate, angular measurements include the UL’s angle of arrival (AoA) and DL’s angle of departure (AoD), distance-based measurements include time of arrival (*ToA*), DL and UL time difference of arrival (*TDoA*), round trip time (RTT) and multi-cell round trip time (MC-RTT). The positioning methods (or estimation algorithms) may be implemented in one or more of the following modes: UE-assisted mode, UE-based mode, stand-alone mode, and network-based mode [14].

New spectrum: Frequency Range 2 (FR2) is an ultrahigh-frequency band allocated for 5G in the millimetre-wave (mmWave) region, spanning from 24.3 GHz to 52.6 GHz. This portion of the spectrum complements the existing Frequency Range 1 (FR1), which covers the bands below 6 GHz and recently expanded to 7 GHz. It addresses the issue of spectrum scarcity in wireless communication systems and enables high data rates, capacity, and bandwidth with low latency, while also providing superior positioning accuracy [3,16]. In this frequency range, radio signals experience penetration and diffraction losses, resulting in a dominant line-of-sight (LOS) component and minimal multipath effects [17]. However, the use of millimeter-wave wireless signals also poses challenges, such as high path loss, which can be mitigated through the adoption of new specialized compensation techniques, such as beamforming and highly directional antennas.

Wideband carriers: NR provides a significant bandwidth improvement over LTE; while LTE provides a maximum of 20 MHz, NR provides up to 100 MHz in Frequency Range 1 (FR1: 450 MHz to 7 GHz) and 400 MHz in Frequency Range 2 [18]. The variance in the delay estimation is inversely proportional to the bandwidth of the signals; this implies that as the signal bandwidth widens, the uncertainty in the delay estimation decreases as the main lobe of the correlation function becomes narrower. Narrower lobes are more easily discriminated by the receiver and, therefore, the distinction between direct and reflected paths improves [19].

Massive multiple-input multiple-output (MIMO) and beamforming: massive MIMO wireless communication refers to the idea of equipping cellular base stations with very large quantities of antennas. Such a massive number of antennas causes interference problems, which can be mitigated by deploying the beamforming technique: a process designed to produce the radiated beam patterns of the antennas by building up the processed signals in the direction of the desired terminals and canceling beams of interfering signals [20]. The benefits of using beamforming in massive MIMO systems include enhanced energy efficiency, improved spectral efficiency, increased system security, and applicability for mmWave bands.

The main purpose of this paper is to implement the positioning using 5G time-of-arrival (*ToA*) observations and to assess the accuracy and the reliability of the estimated coordinates from experimental data. These real-world 5G ranging measurements have been provided by the ESA and were produced during the GINTO5G project, described in the next section. Different strategies to process these data will be compared. In general, when time series of observations are processed to estimate trajectories, a proper filtering process like Kalman filtering [21,22], allows the smoothing of the noise and blunders in individual epochs. On the contrary, we process the epochs independently, using the Least Squares method [23], because we are interested in the accuracy and reliability of the single-epoch solutions, not in the filtering results. In the rapidly evolving field of 5G positioning, the present research introduces an innovative approach in the use of the sounding reference signal. Distinct from the 3GPP specifications for 5G positioning [24], our methodology makes use of the SRS in the downlink direction and proposes signal design changes that would allow it (see Section 2.4 for details). Furthermore, most of the previous research studies on 5G positioning, and in particular positioning using the SRS signal [12,25], are based on simulations rather than real-world experiments carried out in a controlled measurement setup. This setup generates at least two major benefits: repeatability of measurement conditions, and more tangible results than purely theoretical studies. The paper is structured as follows: Section 2 describes the GINTO5G experiment and data collection; Section 3 describes in detail the strategies applied to process the data; Section 4 discusses the results; and finally, Section 5 presents the conclusions.

## 2. The GINTO5G Experiment

As part of ESA’s GINTO5G project, a number of field tests were carried out in 2021 with the aim of collecting and analyzing 5G signals with the specific aim of extracting pseudoranges to estimate the location of the device being tested. For the 5G experiment, an adaptation of the sounding reference signals (SRSs) [24], configured on the downlink instead of the uplink transmission, has been used. This signal was transmitted on 3.7 GHz, spanned 100 MHz in bandwidth, and consisted of a custom frame structure (further details are in Section 2.4). To check the trajectories estimated by 5G the benchmark trajectory was estimated by a total station. It should be specified that although the survey was performed in 2021, this paper summarizes [26] and reports the first experimental results. Technical details of the GINTO5G experiment are described in [27,28,29,30,31] and are just briefly summarized here.

### 2.1. 5G Testbed

The GINTO5G experiment, a collaborative initiative of the ESA with several European companies and universities, represents an important step in validating the potential of 5G for positioning and navigation. A designated testing area was selected to host the specialized infrastructure needed for the experimental 5G signal transmission and reception. This setup is unique in that it is not a conventional operational setup. Instead, it is a self-made engineering construct of a compact 5G private network, tailored specifically for the scope of the GINTO5G project, namely, positioning with 5G signals. The testbed is located at the Fraunhofer Institute in Germany, close to Nürnberg. The measurement setup consists of a transmitting block and a receiving block. The transmitting block is composed of eleven transmitters, paired with an IT ELITE Antenna SEC3710 DP, that emulate a mini 5G private network able to transmit 5G positioning signals. The receiving block consists of a USRP X300 with an omnidirectional antenna programmed to receive 5G signals from the transmitters. Moreover, a Leica MS50 total station is used to survey the ground truth of each trajectory to be estimated.

### 2.2. Experimental Campaign

All measurements were conducted at the L.I.N.K. Test and Evaluation Center of the Fraunhofer IIS campus in Nürnberg (Germany).

**Figure 1:**
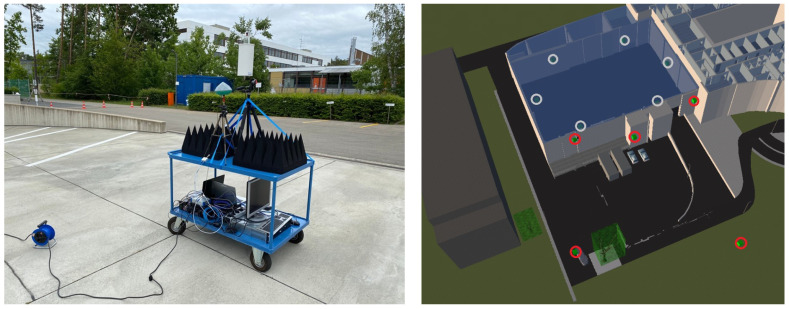
Trolley equipped with USRP for 5G NR downlink measurements (**left**); test center with the 6 indoor (white circles) and 5 outdoor (red circles) TRPs (**right**). Pictures by Fraunhofer IIS.

As shown in Figure 1, eleven transmitters emulating 5G transmitting reference points (TRPs) were distributed across the indoor and outdoor area (loading zone + driveway) and their position was surveyed accurately using a total station prior to the beginning of the 5G data acquisition tests. A trolley was used to host the receiving unit, able to acquire 5G signals, during its movement on a-priori defined trajectories to different areas of the campus. The trolley, shown in Figure 1, was also equipped with a battery backup system and a PC to log the measurements during the field trials.

### 2.3. Execution

The total number of trajectories taken into consideration for this experiment can be clustered into three sub-groups depending on the location of the measurement: loading zone, driveway, or indoor area.

**Figure 2:**
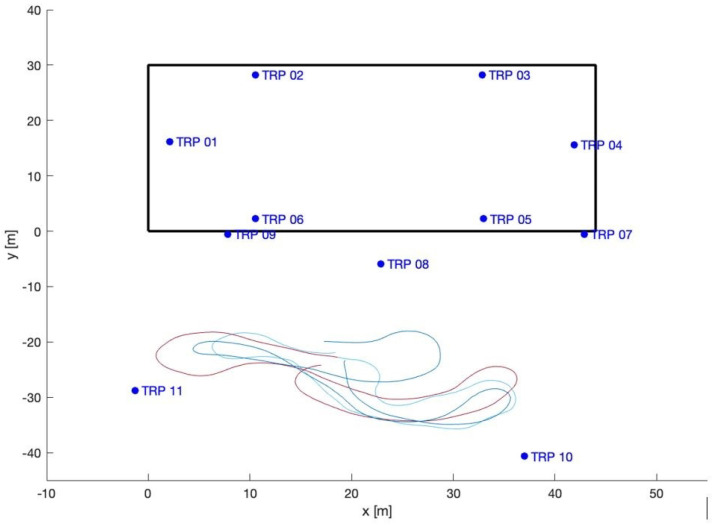
Loading zone trajectories: Takes 01, 02, and 03.

**Figure 3:**
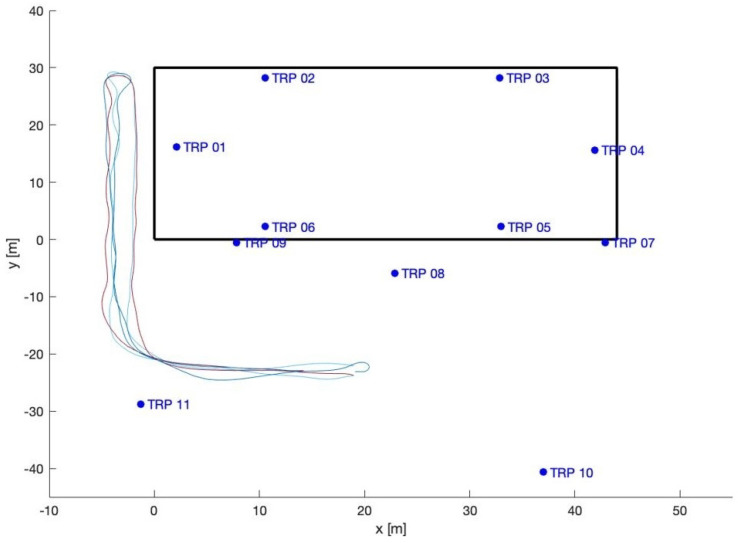
Driveway trajectories: Takes 04 and 05.

**Figure 4:**
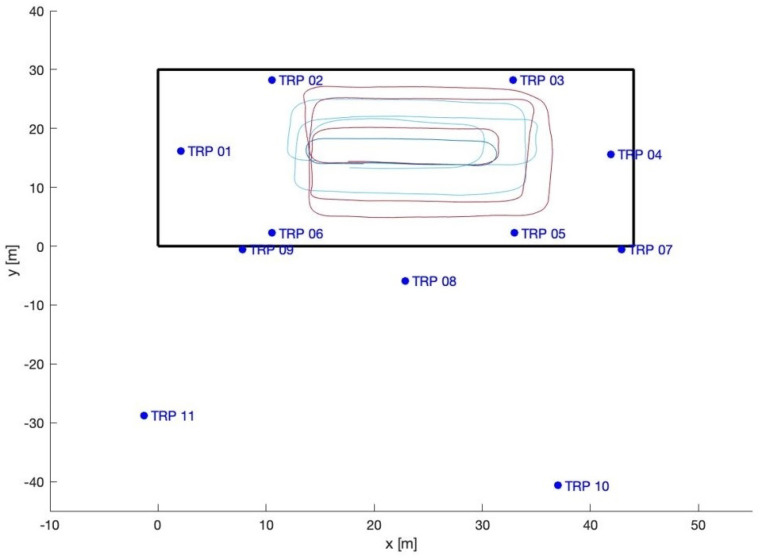
Indoor trajectories: Takes 06, 07, and 08.

- The loading zone (Figure 2), which corresponds to outdoor line-of-sight conditions: Take 01, Take 02, Take 03;
- The driveway (Figure 3), which corresponds to outdoor with a mix of line-of-sight and non-line-of-sight conditions: Take 04, Take 05;
- Indoor area (Figure 4): Take 06, Take 07, Take 08.

### 2.4. Data Recording

This section describes the necessary steps needed to be performed for processing the raw data and, therefore, obtaining *ToA* measurements [27,32,33]. The receiving unit recorded IQ samples generated using the Matlab 5G toolbox and transmitted by the transmitting points.

The computation of 5G NR *ToA* is performed based on the SRSs sent on the downlink channels. This represents an intentional deviation from the 3GPP specifications, which define the use of the SRS on the uplink channel. The choice to adapt the transmission of the SRS to the downlink stems from the fact that existing tools and signal post-processing algorithms were developed for the SRS signal. The adaptation to the SRS signal consisted in prefixing each SRS symbol with a unique secondary synchronization signal (SSS) sequence; this SSS sequence helps to match a specific SRS burst to a transmitting antenna. The SRS signal is transmitted as 10 ms frames, as described in the 3GPP specifications, and its configuration is characterized by a 1 OFDM (orthogonal frequency-division multiplexing) symbol and comb2 transmission pattern with the following bandwidth configuration parameters (Table 1):

This received signal was processed offline as follows: first, the SSS correlation step looked for the maximum SSS peak to allow for different transmitting antennas to be distinguished; followed by SRS correlation based on a fast Fourier transform approach. The maximum SRS correlation peak represents the *ToA* for the signal with respect to the sampling rate. For each 10 ms frame, one *ToA* value was generated.

Although 5G NR transmitters are in general synchronized by a common 10 MHz clock source, the synchronization is not perfect. There is a delay caused by a number of factors, including different cable lengths and the connection between the antenna and ADC (analog to digital converter), and it needs to be compensated for. Furthermore, additional, non-constant latency can be introduced by each USRP on start-up, necessitating the performance of latency compensation at least once each time the setup is turned on. The following equation gives the effect of latency on the *ToA* measurement:

(1) $$ToA_{R}^{BS}=\tau_{R}^{BS}+dt_{R}-dt^{BS}$$

where τ is the time of flight of the signal from the base station, and $dt_{R}$ and $dt_{BS}$ are, respectively, the clock offset of the receiver and of the base station with respect to a given reference time scale. In the GINTO5G experiment, all the base stations were electronically synchronized and the residual synchronization error resulted in post-set-up calibration with a magnitude of a few meters, which was used to correct the measured *ToA*. This instrumental calibration is not discussed here because it was preliminary to this work [31]. As a result, the *ToA* contains just the geometry and the clock offset of the receiver. As further detailed in Section 3, the receiver clock offset was removed by time differencing pairs of *ToA* measurements, leading, therefore, to *TDoA* measurements. The calibrated observations contain errors that can be ascribed to three different sources:

- clock jitter;
- radio channel effects (e.g., multipath);
- the accuracy of the reference measurements of the distances between antennas.

Although clock jitter has a general impact on the *ToA* quality, it cannot be directly compensated for, and in general, low-jitter clocks (picosecond jitter) have to be taken into account during system design. The equipment and conditions used in this work were experimental and well controlled, a situation difficult to reproduce on a standard mass-market device: picosecond-jitter clocks indeed suggest that such results cannot currently be reproduced with mass-market devices/smartphones. This clearly will be an object of future investigations. The coordinates of the base stations and the reference trajectories are given by a terrestrial survey with a Leica MS50 total station: considering the used instrumentation and the surveying technique, we can assume that the accuracy of the benchmark results is at the centimeter level, at least a magnitude better than the FR1 accuracy. As a result, the main error sources in the measurements are channel effects like multipath, and accordingly measuring line-of-sight signals is crucial for a low-error calibration.

## 3. Methods

**Figure 5:**
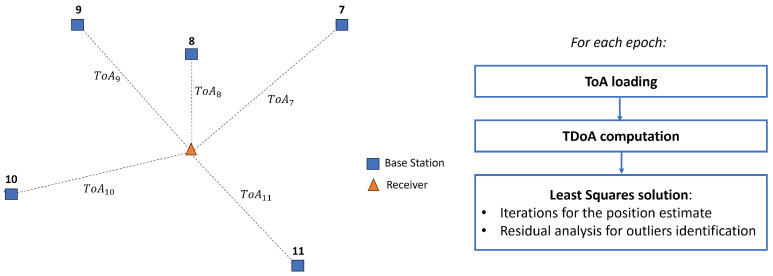
*ToA* observations (**left**); processing flux diagram (**right**).

The section focuses on a description of the algorithms implemented to process the *ToA* observations. The available GINTO5G datasets include 5G time of arrival (*ToA*) observations (Figure 5, left), their signal-to-noise ratio (SNR) values, local Cartesian coordinates of the base stations, and the ground truth output from the total station. In the processing, the time difference of arrival (*TDoA*) values will be utilized as the observations. As specified in Section 2, the underlying assumption is that all the base stations are synchronized; hence their clock offsets are set to zero. The clock term of the receiver can be eliminated by differentiating two simultaneous *ToA* observations. In fact, given the receiver *R* and the two *ToA* to two base stations

(2) $$ToA_{R}^{BS_{i}}\left(t\right)=\tau_{R}^{BS_{i}}\left(t\right)+dt_{R}\left(t\right),ToA_{R}^{BS_{j}}\left(t\right)=\tau_{R}^{BS_{j}}\left(t\right)+dt_{R}\left(t\right).$$

choosing BSi as the reference station, the relevant *TDoA* is given by

(3) $$TDoA_{i,j}\left(t\right)=\tau_{R}^{BS_{i}}\left(t\right)-\tau_{R}^{BS_{j}}\left(t\right),$$

Multiplying by the signal velocity *c*, the observed *ToA* can be converted to metric pseudoranges:

(4) $$c\cdot ToA_{R}^{BS_{i}}\left(t\right)=\rho_{R}^{BS_{i}}\left(t\right)+c\cdot dt_{R}\left(t\right),$$

where $\rho_{R}^{BS_{i}}$ is the geometric distance between the base station and the user receiver. Note that the differences of the metric observations, $c\cdot TDoA_{i,j}$, are directly differences of distances.

From a geometrical point of view, the pre-elimination of the clock in the *TDoA* values does not present any specific advantage with respect to the processing of undifferenced observations (see, for example, [34]); indeed, it is counter balanced by the reduction in the input observations. However, we decided to process the *TDoA* to investigate this specific approach, and also because it is very popular in the technical literature. Given these premises, our objectives in this study are:

1. Compute the *TDoA*;
2. Investigate possible strategies for the choice of the reference station in the *TDoA* (Section 3.1);
3. Estimate the positions using the least squares method in single epochs (Section 3.2);
4. Analyze the results and the statistics of the estimated trajectories with respect to the ground truth (Section 4).

All the base stations of the experiment are at the same height, which is also approximately the height of the trolley. As was preliminarily discussed in the technical documentation of GINTO5G, with such a configuration, the estimation of the vertical coordinate causes an ill conditioning of the resulting system; therefore, only horizontal coordinates will be estimated.

### 3.1. TDoA Analysis

In order to verify the accuracy and reliability of the 5G observations, the measured *TDoA* are compared with the reference *TDoA* obtained from the known distances between the base stations (*BS*s) and the receiver, as determined by the coordinates provided by the total station benchmark *TDoA*).

In each trajectory, different approaches are tested for choosing the reference station for *TDoA* computation:

- use one *BS* as reference for all the epochs;
- use the pivot method, e.g., following a scheme *BS*1–*BS*2, *BS*2–*BS*3, and so on, for each epoch;
- choose as reference station for each epoch the station with the best SNR: in this case, the configuration can change between epochs.

It is worth noting that, in pivoting, the selection of a specific scheme is not important. Indeed, by a proper propagation of the covariance matrix (as in Equation (10)), the solution of the least squares method for different pivoting strategies should be identical up to numerical rounding [34]. The results of this analysis are discussed in Section 4.1.

### 3.2. Least Squares Algorithm

In the single-epoch solution, the unknowns are the horizontal coordinates of the receiver [$x_{R},y_{R}$], while the height is kept constant.

Since the least squares method is applicable only to a set of linear equations, the processing involves the linearization of the observation equations with respect to the receiver’s coordinates. The starting point of the process is the general formula for the distance between BS and *R*:

(5) $$\rho_{R}^{BS}={\tilde{\rho }}_{R}^{BS}+{\tilde{e}}_{R}^{BS}\cdot {\mathrm{dx}}_{R}$$

where ${\tilde{\rho }}_{R}^{BS}$ and ${\tilde{e}}_{R}^{BS}$ are, respectively, the approximated distance and the unitary vector from BS to *R*; ${\mathrm{dx}}_{R}$ is the difference between the true and the approximated coordinates of *R*. Considering two base stations (*BS*1 and *BS*2), and remembering that the *TDoA* will be the input of the algorithm, the difference in the distances can be written as

(6) $$\rho_{R}^{BS1,BS2}={\tilde{\rho }}_{R}^{BS1,BS2}+\Delta e_{R}^{BS1,BS2}\cdot {\mathrm{dx}}_{R}$$

where $\rho_{R}^{BS1,BS2}$ is the difference in the two distances, from the two BS to *R*. $\Delta e_{R}^{BS1,BS2}$ is the difference of the two unitary vectors, from BS1 and BS2 to *R*.

Given all the available *n* *ToA* in one epoch, n−1 *TDoA* can be derived. A system can be written introducing the classical notation of the least squares method:

(7) $$y=A\cdot {\mathrm{dx}}_{R}+b$$

where y is the vector of the observables, b is the vector containing the known terms, and A is the design matrix, whose n−1 rows are simply the transpose of $\Delta e_{R}^{BSi,BSj}$.

The final LS solution is given by

(8) $${\hat{\mathrm{dx}}}_{R}=N^{-1}A^{T}Q^{-1}\left(y_{0}-b\right)$$

where Q is the cofactor matrix of *TDoA*, $y_{0}$ are the available observations, and $N=A^{T}Q^{-1}A$. In addition to the unknowns, their covariance matrix can be estimated:

(9) $$C_{xx}={\hat{\sigma }}^{2}N^{-1}$$

where ${\hat{\sigma }}^{2}$ is the a posteriori variance. Even the observations’ residuals and normalized residuals can be computed according to [23].

Q is obtained as follows, given the cofactor matrix C of the undifferenced *ToA*:

(10) $$Q=\Delta C\Delta^{T}$$

where Δ is the matrix of the linear transformation from *ToA* to *TDoA*. In our specific case, arranging the stations in order to have the reference one in the first position:

(11) $$\Delta =\left[\begin{array}{ccccc} 1 & -1 & 0 & \cdots & 0\\ 1 & 0 & -1 & \cdots & 0\\ \cdots & \cdots & \cdots & \cdots & \cdots \\ 1 & 0 & 0 & \cdots & -1 \end{array}\right],$$

We assume that the available *ToA*s are not correlated: therefore, when weights are applied, C is defined as

(12) $$C=\left[\begin{array}{ccccc} \eta^{BS1} & 0 & 0 & \cdots & 0\\ 0 & \eta^{BS2} & 0 & \cdots & 0\\ \cdots & \cdots & \cdots & \cdots & \cdots \\ 0 & 0 & 0 & \cdots & \eta^{BSn} \end{array}\right]$$

where the weights can be, for example, given by

(13) $$\eta^{BSi}=\frac{1}{SNR_{i}^{2}}.$$

In case weights are not applied, each η=1. The weighting of the *ToA* will be discussed in the following sections. Figure 5 (right) shows the LS processing scheme.

Linearized least squares requires iterations: in our application, the initial approximate coordinates are set in the barycenter of the base stations, at each following iteration the estimated values at the previous iteration are used. The algorithm iterates up to a maximum number of 20 iterations, unless the convergence (set to one millimeter) is reached.

To assess the accuracy of the solutions, for each epoch the estimated position is compared with the ground truth:

(14) $$\epsilon_{x}\left(t\right)={\hat{x}}_{5G}\left(t\right)-x_{TS}\left(t\right)$$

(15) $$\epsilon_{y}\left(t\right)={\hat{y}}_{5G}\left(t\right)-y_{TS}\left(t\right),$$

(16) $$\epsilon_{2D}\left(t\right)=\sqrt{\epsilon_{x}{\left(t\right)}^{2}+\epsilon_{y}{\left(t\right)}^{2}}.$$

where *t* is the epoch, $\left[{\hat{x}}_{5G},{\hat{y}}_{5G}\right]$ is the estimated position, $\left[x_{TS},y_{TS}\right]$ is the position from the total station. The average and standard deviation of the errors for *x* and *y* as well as the 2D plane are then calculated for each trajectory:

(17) $$\mu_{\epsilon }=\frac{1}{N}\sum_{t=0}^{N}\epsilon_{2D}\left(t\right)$$

(18) $$\sigma_{\epsilon }=\sqrt{\frac{\sum_{t=0}^{N}\epsilon_{2D}{\left(t\right)}^{2}}{N-1}}.$$

where *N* is the number of epochs. This analysis helps to identify any patterns in the errors and provides a better understanding of the accuracy of the 5G trajectory. Least squares convergence and the final results are discussed in Section 4.2.

## 4. Results

### 4.1. SNR Analysis

A preliminary analysis of the SNR values is performed. We, firstly, discuss the findings obtained from analyzing Take 01.

**Figure 6:**
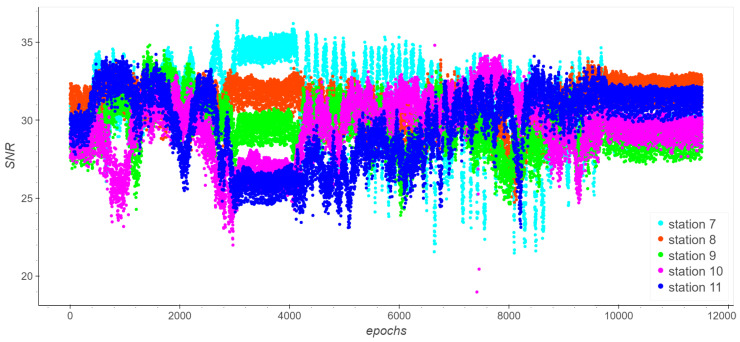
Take 01. SNR (dB) values plotted against time for each base station.

We investigate which base station has the best mean SNR value and how the distance between the base station and the trolley can influence it. A graph illustrating the SNR values for all the outdoor stations is shown in Figure 6.

**Figure 7:**
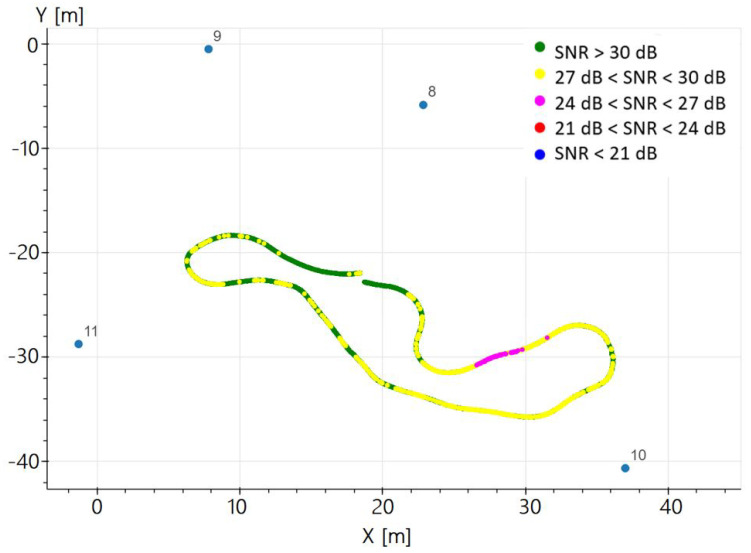
Take 01, base station 8: SNR along the trajectory.

**Figure 8:**
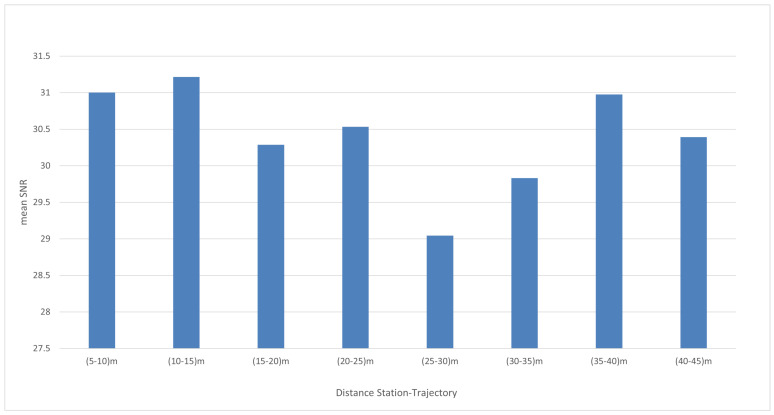
Take 01: mean SNR for each distance interval.

The constant or almost constant values correspond to static or low straight dynamic intervals. Stations 7 and 8 provide the best SNR values. Statistics of the SNR values are computed for each station (Table 2). As an example, Figure 7 depicts the SNR of station 8 along the trajectory, while Figure 8 displays the mean SNR within each distance interval for all the stations.

For our data, no correlation exists between the SNR values and the distances between the stations and the receiver. This is due to the small range of distances and covered area. The objective of the preliminary SNR investigation is to search for a criterion to select the optimal reference station for the *TDoA*. In the following, we assess the influence of the SNR of the reference station on the quality of the data. For each take, the following scenarios are considered for the *TDoA* computation:

- one reference station for the whole trajectory (this choice is repeated for each one station);
- the pivot method, with the schema: *BS*7–*BS*8, *BS*8–*BS*9, *BS*9–*BS*10, *BS*10–*BS*11;
- at each epoch the reference station is the station with the best SNR.

For all the above cases we compute the *TDoA* individual errors, as the differences between the observed *TDoA* and the true single differences of the distances between the stations and the receiver. Table 3 shows the statistics on the *TDoA* errors for each case. In the analysis of the errors, a few observations of some stations are clearly affected by isolated blunders of more than 100 m. These observations, less than ten, are excluded by the following analysis and least squares solutions.

In our data, no correlation exists between the SNR of the reference station and the resulting *TDoA* errors; therefore, the SNR sorting is not an optimal criterion to select the best reference station in *TDoA* computation. The above conclusions, discussed in detail, are relevant to Take 01, but all the other takes provide similar results.

### 4.2. Least Squares Solution

In this section, we will present the positions and trajectories estimated using the least squares method in a single epoch utilizing the *TDoA*s as inputs. The presentation of the results will start with the trajectories in the loading zone, followed by the indoor trajectories, and conclude with the trajectories in the driveway zone. Taking into account the results of the previous section, no SNR weighting is applied to the observations. Moreover, each trajectory is estimated by using one reference station for all the epochs. In the least squares iterations, we anticipate that in all the processing the convergence is reached in less than five iterations.

#### 4.2.1. Loading Zone Trajectories: Takes 01, 02, and 03

**Figure 9:**
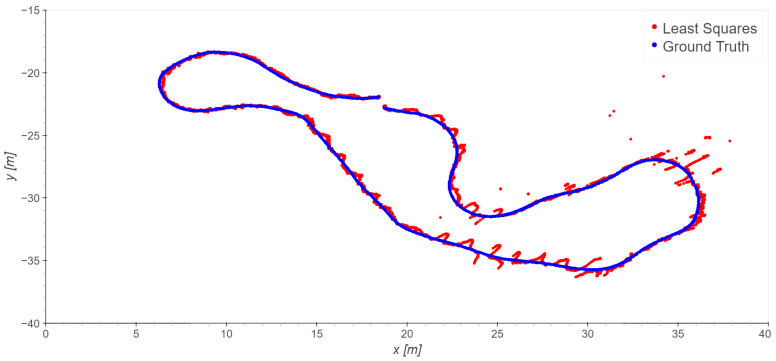
Take 01, reference station 8: least squares results.

**Figure 10:**
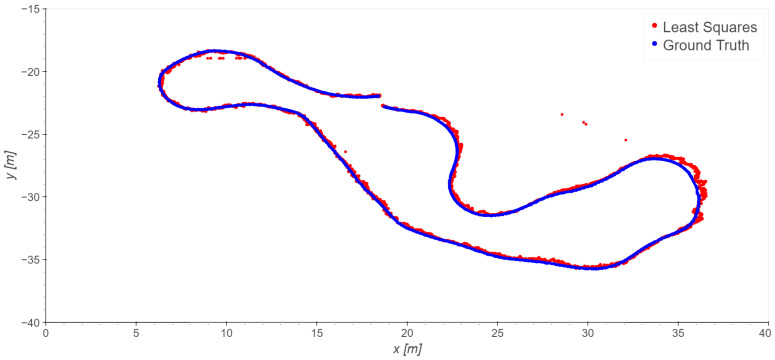
Take 01, reference station 8: least squares results excluding station 7.

In the loading zone, only the outdoor stations (7, 8, 9, 10, 11) are used. In this area, a good line of sight (LOS) is expected. The trajectory of Take 01 is analyzed first. The trajectory is repeatedly estimated by using as the *TDoA* reference, firstly, station 7, then 8, and so on, up to 10. By a visual check of the results and the residuals, the observations of station 7 are clearly affected by the largest errors, both locally correlated and sparse outliers. This is probably due to the presence of a reflecting surface near the station, and the resulting multipath. By excluding station 7 from the dataset, the results clearly improve, due to a significant reduction in spurious patterns and isolated outliers; compare, for example, Figure 9 and Figure 10. Considering that, in general, station 7 showed the best SNR, this confirms that in our data the SNR does not provide a useful index of quality.

The final statistics of Take 01 excluding station 7 are shown in Table 4 for different choices of reference stations; the results are almost homogeneous.

**Figure 11:**
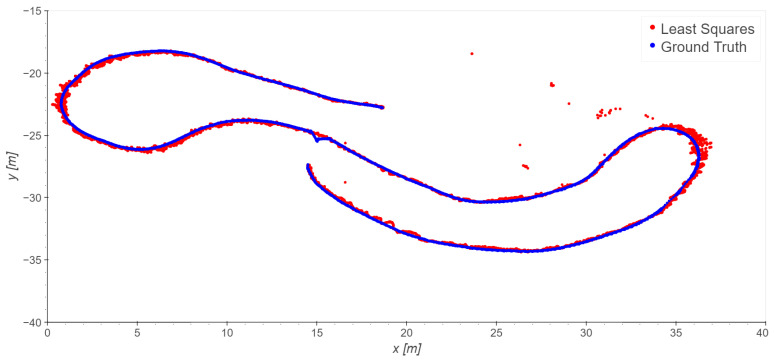
Take 02, reference station 8: least squares results excluding station 7.

**Figure 12:**
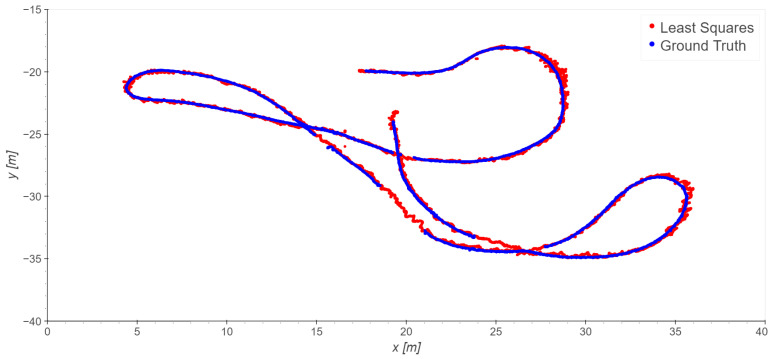
Take 03, reference station 8: least squares excluding station 7. Note: for a few epochs, the total station did not record measurements; these epochs are excluded from any statistical analysis.

Similar results have been obtained for Takes 02 and 03; the exclusion of station 7 improves the statistics in these cases as well. The results of using least squares on Takes 02 and 03 are presented in Figure 11 and Figure 12 and Table 5. Note that in Take 02 the worst results are in the same area as those of Take 01.

Note that the exclusion of station 7 locally improves the quality of the results but does not significantly change the general statistics. After removing station 7, the redundancy of the the least squares method estimates in single epochs is very low (one, equal to three *TDoA* observations minus two unknowns).

#### 4.2.2. Indoor Trajectories: Takes 06, 07, and 08

**Figure 13:**
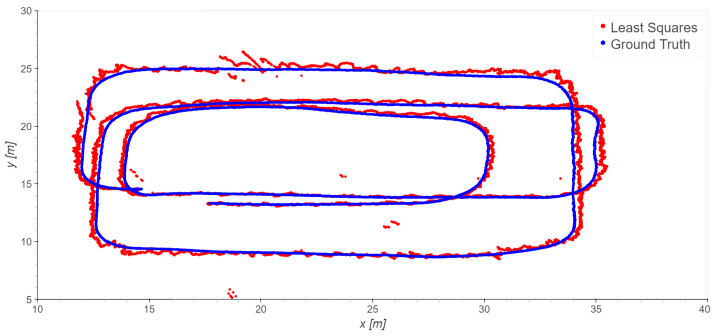
Take 06, reference station 1: least squares results.

**Figure 14:**
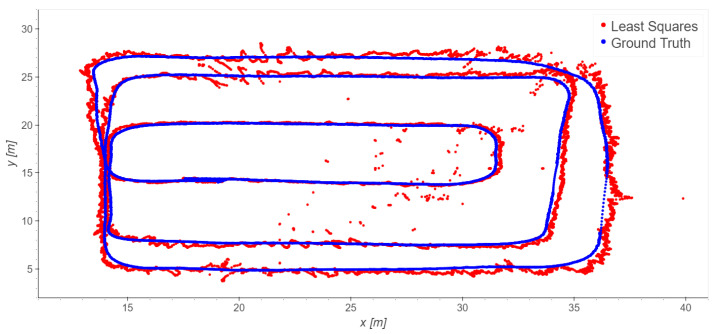
Take 07, reference station 1: least squares results.

**Figure 15:**
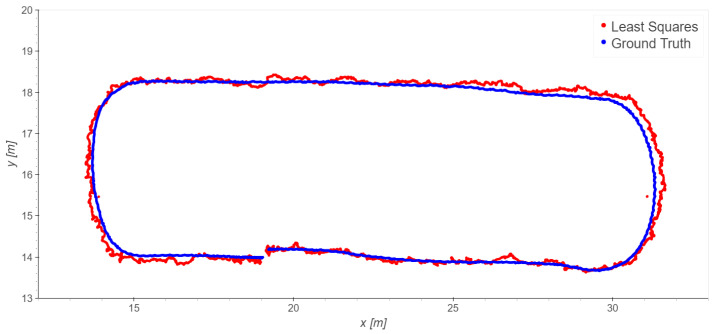
Take 08, reference station 1: least squares results.

For indoors, we consider the six indoor base stations (1, 2, 3, 4, 5, 6). In this case, different choices of the reference station were also tested; however, none of them significantly outperforms the others in term of the error statistics. Therefore, to avoid repetition, we present just the results obtained using station 1 as the reference (Figure 13, Figure 14 and Figure 15 and Table 6).

Take 08 exhibits excellent results, while Take 06 shows a slightly worse performance, and Take 07 provides the worst results. In any case, the indoor results are generally good considering all the disturbances, mainly interferences and multipath, that can occur in such scenarios. Blunders are present in single epochs; as premised in the introduction, they could be smoothed using time-series filtering, but that is outside the scope of the present paper. Table 7 presents an analysis of the residuals obtained using the least squares method for all the trajectories. The observations of *TDoA*s *BS*1–*BS*2 provide the worst results: this is particularly significant in Take 06, while in the other takes the residuals are more homogeneous.

As for the loading zone, we remove the data of the worst station, in this case station 2. Table 8 and Table 9 report the statistics of Take 06, which improve significantly; the other takes do not significantly improve.

#### 4.2.3. Driveway Trajectories: Takes 04 and 05

In the driveway processing, only the outdoor base stations were used. The driveway trajectories start at the loading zone, where LOS is available for all the stations, then the trajectories move to NLOS conditions.

**Figure 16:**
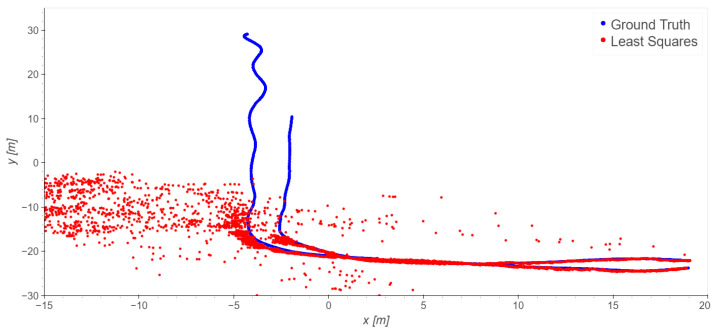
Take 04, reference station 4: least squares results.

**Figure 17:**
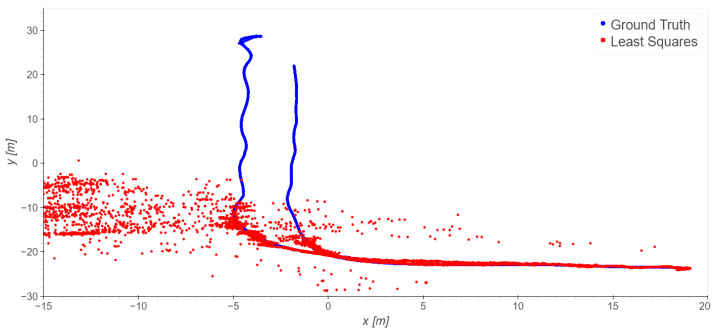
Take 05, reference station 4: least squares results.

**Figure 18:**
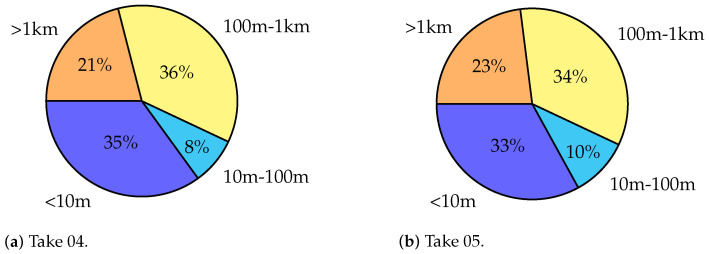
Pie charts representing the error magnitudes in Takes 04 and 05.

The positioning (Figure 16 and Figure 17) along the north-west part of the trajectories suffers from shadowing; once the trolley moves closer to the building and enters a non-line-of-sight (NLOS) condition the solutions are completely blinded and meaningless. As the usual statistics of errors (mean, standard deviation, …) are useless, Figure 18 displays the percentages of the errors’ magnitudes. The results are very bad and not usable in practice, but they were expected because of the NLOS conditions of this scenario.

## 5. Conclusions

This experiment provided the opportunity to accomplish the following aims:

- to implement algorithms for positioning using 5G observations that were applied to 5G data from the GINTO5G experiment;
- to experimentally assess the accuracy of the positioning in an environment where the deployment of the base stations was carefully controlled and optimized;
- to conduct experimental research on techniques aimed at identifying and reducing measurement errors.

The processing focused on *TDoA* observations, processed in a single epoch using the least squares method. The accuracies of the estimated positions reach the decimeter and the meter levels, respectively, in outdoor and indoor scenarios. Within this specific experiment, the SNRs of the base stations do not exhibit a significant correlation with distance with respect to the receiver; moreover, the use of the SNR as a criterion to choose the reference station in *TDoA* does not improve the quality of the differenced observations. This can be explained by the fact that the GINTO5G experimental area was relatively small, spanning 40 m × 40 m; moreover, the data of the experiment were calibrated in order to remove the clock biases of the base stations, and this explains the good quality of the results. In our scenario, the redundancies of the single-epoch solutions are small. Some outliers exist when obstructions and reflecting surfaces are present, especially indoors. Clearly, with larger redundancies in single epochs, or by time-series filtering, the estimates could improve. In any case, the obtained results in LOS have accuracies that range from decimeters to meters.

Given these experimental premises, the achieved results are satisfying and promising for further research about mass-market applications. In conclusion, the hybridization of 5G and GNSS signals for positioning holds great promise for a wide range of applications, from autonomous vehicles and augmented reality to smart cities and beyond. 

## Figures and Tables

**Table 1:** Bandwidth configuration parameters, as specified in [24].

Parameter	Value
$C_{SRS}$	63
$B_{SRS}$	0
$m_{SRS}$	272

**Table 2:** Take 01: statistics of SNRs (dB) of the base stations.

	*BS* 7	*BS* 8	*BS* 9	*BS* 10	*BS* 11
Mean (dB)	31	31	30	30	30
St.dev. (dB)	2	1	1	2	2
Max. (dB)	36	34	35	35	34
Min. (dB)	22	25	24	19	23

**Table 3:** Take 01: errors (measured minus ground truth) in *TDoA* for different choices of reference station. Ref. i: base station i as reference for all the epochs; Pivot: pivot scheme; Best SNR: for each epoch the best SNR at each epoch. Mean: mean; St. dev.: standard deviation. Cum. err.: cumulative error, square root of the sum of the squares of the errors divided by the population.

	Ref. [7]	Ref. [8]	Ref. [9]	Ref. [10]	Ref. [11]	Pivot	Best SNR
Cum. err. (m)	0.8	0.6	0.8	1.0	0.8	0.9	0.8
Mean (m)	0.1	−0.1	0.0	0.5	−0.2	0.0	0.2
St. dev. (m)	0.6	0.4	0.5	0.6	0.6	0.7	0.7

**Table 4:** Take 01: statistics of 2D errors for each reference station after excluding station 7.

2D Error	Ref. [8]	Ref. [9]	Ref. [10]	Ref. [11]
Mean (m)	0.6	0.6	0.6	0.6
St. dev. (m)	0.4	0.4	0.4	0.4
Max. (m)	7.2	6.9	7.3	7.4

**Table 5:** Takes 02 and 03, reference station 8. Statistics of 2D errors with and excluding station 7. >10 m, >5 m: number of observations with residuals bigger than 10 m and 5 m, respectively.

2D Error	Mean (m)	St.dev. (m)	Max. (m)	>10 m (n)	>5 m (n)
Take 02 with st. 7	0.9	1.0	29	10	20
Take 02 excl. st. 7	0.8	0.9	27	9	15
Take 03 with st. 7	0.9	0.7	5	2	2
Take 03 excl. st. 7	0.9	0.6	3	2	2

**Table 6:** Takes 06, 07, and 08, reference station 1. Statistics of 2D errors.

2D Error	Mean	St. dev.	Max.
Take 06 (m)	0.7	0.8	4.9
Take 07 (m)	1.9	1.9	19
Take 08 (m)	0.6	0.4	1.6

**Table 7:** Takes 06, 07, and 08, reference station 1. Statistics of the 2D *TDoA* residuals.

Residuals		1–2	1–3	1–4	1–5	1–6
Take 06	Mean (m)	−0.1	0.0	−0.1	−0.1	−0.1
	St. dev. (m)	1.6	0.2	0.6	0.9	0.6
	Max. (m)	39	3	14	21	17
Take 07	Mean (m)	0.04	−0.2	0.1	−0.02	−0.1
	St. dev. (m)	1.5	1.3	0.9	0.9	0.8
	Max. (m)	37	25	15	23	15
Take 08	Mean (m)	0.0	0.1	0.1	0.0	0.2
	St. dev. (m)	0.2	0.1	0.1	0.1	0.2
	Max. (m)	0.5	0.6	1.0	0.5	0.7

**Table 8:** Take 06, reference station 1. Statistics of 2D errors excluding station 2.

Mean	0.6 m
St. dev.	0.4 m
Max.	1.8 m

**Table 9:** Take 06, reference station 1. Statistics of *TDoA* residuals excluding station 2.

Residuals	1–3	1–4	1–5	1–6
Mean (m)	0.0	0.0	0.0	0.0
St. dev. (m)	0.1	0.1	0.1	0.2
Max. (m)	0.7	0.6	0.6	0.7

## Abbreviations

The following abbreviations are used in this manuscript:

GNSS	Global navigation satellite system
*ToA*	Time of arrival
*TDoA*	Time difference of arrival
LOS	Line of sight
NLOS	Non-line of sight
*BS*	5G base station
UE	User equipment
SNR	Signal to noise ratio
SRS	Sounding reference signal
SSS	Secondary synchronization signal
OFDM	Orthogonal frequency-division multiplexing
